# Social and Poor-Law Problems

**Published:** 1908-05-30

**Authors:** 


### SOCIAL AND POOR-LAW PROBLEMS.

### ELLINOR HOME FARM, NEW BRUNSWICK.

It is about two years since Mrs. Ellinor Close brought forward her scheme for transferring Poor-law children to Canada, to be brought up there. The *Times* lately published a long account, from a correspondent, of the home which she has established in New Brunswick, where twelve children—ten boys and two girls whose ages range from six to fifteen—are being brought up. The account is highly laudatory. It tells how children, brought "thin and anæmic" from city slums in England, have grown robust-looking in the western air. It also records that the Government of New Brunswick looks favourably on the scheme, and the neighbourhood is sympathetic towards it. Finally it says that the capital cost works out at £75 per child, and maintenance charges at £22 per head per annum.

These are the simple facts, spread out through a column and a half of eulogistic comment. Doubtless it is all true, but, even so, what is there in the scheme that is either new or wonderful? The home is, in Poor-law phrase, a "scattered home." We have them in England, and here also thin and anæmic children grow robust and rosy. Here, also, the local authorities are not unfavourable, and the neighbourhood offers no objection to the establishment of a home whose inmates will consume their meat and bread and groceries. The advantages are entirely on the side of the neighbourhood where the home is established, and either in New Brunswick or any other place, people welcome customers who give good orders and whose money is sure. If Mrs. Close wants to give a home in New Brunswick or anywhere else in the healthier parts of the Empire to waifs and strays, as Dr. Barnardo did, and if she can, like him, get private persons to support her in her scheme, we will wish her every success.

But when she wishes to get Poor-law children for her colonists, she introduces a new element into the question. THE HOSPITAL has always been strongly in favour of the boarding-out of Poor-law children, being convinced that in this we find the nearest approach to normal home life to which these unfortunates can attain. But it must be boarding-out under the convenient or at least possible supervision of the guardians who are responsible for the welfare of these children. Should we say that the guardian of any ordinary child was doing his duty if he sent the child to a school several thousands of miles away? The guardian of the Poor-law child has the same responsibilities and should not, if he can help it, send his ward so far away that he cannot inform himself of his welfare by personal investigation. Pecuniarily the scheme has no advantages for the ratepayer—and the man who pays has a right to be considered so long as his natural desire for economy does not clash with the interests of his compulsory protégés. A child can be boarded out in a cottage home for from £12 to £15 per annum, so why should one pay £22 (not including the £75 per head capital expenditure) for the privilege of having him brought up in New Brunswick? If a farm colony is wanted by any Union, there would be little difficulty in getting the necessary land, or the "two English ladies, one of them a trained nurse," who take care of the children in Canada. In short, there are no advantages in the plan which would not be equally attainable in England, and the question is whether, for at best equal advantages, it is worth while spending the ratepayers' money in New Brunswick instead of in England.

Moreover, the scheme at best could apply only to a few of the wards of the State—namely, to orphans and those children who, because of the bad character of their natural protectors, are "adopted" by the guardians. But a very large proportion of the Poor-law children are simply fatherless, and though their mothers require State aid to bring them up they do not relinquish all claim to them. And anyone with even the slightest Poor-law experience knows how difficult it is to get the consent of the mother to the apprenticing of even big boys and girls a hundred miles out of the Union, however promising the prospects of the child might be if thus sent away. Is it likely that these same women, affectionate but ignorant, would consent to the emigration of their children to Canada? We believe that in the first draft of Mrs. Close's scheme it was planned that the children, having been brought up in Canada, should be, when their education was completed, brought back to England and put to work here. Perhaps this suggestion was made to reconcile the Unions here to bearing the cost of the rather expensive maintenance of the children through their helpless years by the prospect of their work being a national asset when their education was concluded. We doubt much if children brought up in Canada would make themselves at home again in the Old Country, and as for the "adopted" children who are among the few for whom the scheme is available, the great aim is to keep them away from the evil influences which surrounded their infancy at the impressionable years when they are entering upon man- and womanhood, it is distinctly undesirable that they should. We believe strongly in emigration, but it should be the permanent emigration of boys and girls who are of an age to begin work, and whose constitutions have been proved to be vigorous enough for the undeniably hard conditions of Canadian life, not for young children who could be as well provided for at home; and certainly they should not be sent abroad with the idea of bringing them back in their teens to a country with whose habits of living they are no longer in sympathy. If the Governments of New Brunswick or of any other part of the Dominion of Canada want English Poor-law children, it is open to them to place proposals before the Local Government Board or before any of our Unions, showing what they propose to do for them when they get them across the Atlantic. While there may be no objection to Mrs. Close's scheme, considered as a voluntary effort, supported by herself and her friends for such children as they may choose to send, it is not one on which boards of guardians would be justified in spending money drawn compulsorily from the ratepayers.

